# Stability of ^47^Sc-complexes with acyclic polyamino-polycarboxylate ligands

**DOI:** 10.1007/s10967-012-2188-x

**Published:** 2012-09-13

**Authors:** Magdalena Połosak, Agata Piotrowska, Seweryn Krajewski, Aleksander Bilewicz

**Affiliations:** Institute of Nuclear Chemistry and Technology, Dorodna16, 03-195 Warsaw, Poland

**Keywords:** Scandium radionuclides, Targeted therapy, Acyclic multidentate ligands

## Abstract

The aim of this study was to evaluate acyclic ligands which can be applied for labeling proteins such as monoclonal antibodies and their fragments with scandium radionuclides. Recently, scandium isotopes (^47^Sc, ^44^Sc) are more available and their properties are convenient for radiotherapy or PET imaging. They can be used together as “matched pair” in theranostic approach. Because proteins denaturize at temperature above 42 °C, ligands which efficiently form complexes at room temperature, are necessary for labelling such biomolecules. For complexation of scandium radionuclides open chain ligands DTPA, HBED, BAPTA, EGTA, TTHA and deferoxamine have been chosen. We found that the ligands studied (except HBED) form strong complexes within 10 min and that the radiolabelling yield varies between 96 and 99 %. The complexes were stable in isotonic NaCl, but stability of ^46^Sc-TTHA, ^46^Sc-BAPTA and ^46^Sc-HBED in PBS buffer was low, due to formation by Sc^3+^stronger complexes with phosphates than with the studied ligands. From the radiolabelling studies with n.c.a. ^47^Sc we can conclude that the most stable complexes are formed by the 8-dentate DTPA and EGTA ligands.

## Introduction

Radionuclides emitting low and medium energy beta-particles and having several days half-life are attractive candidates for radioimmunotherapy. The most promising radioisotope in this category is ^177^Lu, which has favourable decay characteristics, as e.g. half-life of 6.71 days.

This radionuclide can be produced in nuclear reactors by *n*,γ reaction and is commercially available at high levels of specific activity and chemical purity.

Having large cross-section of 2090 b [1], ^177^Lu can be directly produced with a relatively high specific activity by neutron activation of ^176^Lu (2.6 % of natural abundance). Nevertheless, some amount of stable ^176^Lu cannot be avoided, which may cause some problems concerning receptor saturation with biomolecules labelled with stable isotope, especially when the number of receptors is limited. For this purpose an alternative production route via neutron capture, starting with enriched ^176^Yb targets, has been demonstrated [2–4]. In this method, the isotopically enriched ^176^Yb target undergoes the (*n*,γ) reaction to produce ^177^Yb, which subsequently decays by β^−^-emission (*T*_1/2_ = 1.9 h) to ^177^Lu. However, this indirect production route requires difficult radiochemical separation of ^177^Lu from the irradiated Yb_2_O_3_ target. Taking into account that ytterbium and lutetium are neighbouring trivalent lanthanides and that the Yb/Lu mass ratio in the irradiated target can be as high as several thousand, separation is a very difficult task [2, 5, 6].

The usefulness of other radionuclides, which may enhance the therapeutic effect of radiopharmaceuticals, should be also investigated. As a good alternative to application of carrier-free ^177^Lu Lehenberger et al. [7] proposed application of reactor produced ^161^Tb. The properties of this radionuclide are similar to those of ^177^Lu, but separation from the target is considerably easier.

Application of ^47^Sc, as an alternative radionuclide to ^177^Lu, was proposed in earlier works [8–11]. ^47^Sc is a low energy β^−^-emitter with a 3.35 days half-life, and shows a primary γ-ray at 159 keV, which is suitable for imaging. It is important to mention that other scandium radionuclides, ^43^Sc and ^44^Sc, are also promising β^+^-emitters, suitable for PET technique. Therefore, therapeutic ^47^Sc together with diagnostic ^44^Sc or ^43^Sc can be used as “matched pair” in teranostic approach. Essential decay characteristics and production parameters for n.c.a. ^47^Sc, ^177^Lu and ^161^Tb are presented in Table 1.

**Table 1:** Production parameters of n.c.a. ^177^Lu, ^161^Tb and ^47^Sc

Radionuclide	T_1/2_ (days)	Nuclear reactions	E_βmax_ (MeV)	Main photons, keV (%)	Cross-section (barn) (Vertes 2011)	Cost of enriched target ($/mg)	Separation from the target
^177^Lu	6.647	^176^Yb(*n*,γ)^177^Yb $$ \underrightarrow {\beta {\kern 1pt} - } $$ ^177^Lu	0.14	54 (33.5)	3	13	Very difficult
				208.4 (60.9)			
				229 (37.2)			
^161^Tb	6.906	^160^Gd(*n*,γ)^161^Gd $$ \underrightarrow {\beta {\kern 1pt} - } $$ ^161^Tb	0.15	25.7 (21)	1.5	5	Difficult
				48.9 (16)			
				74.6 (9.8)			
^47^Sc	3.341	^47^Ti(*n*,p)^47^Sc	0.16	159.4 (68)	0.15	10	Easy

The production method of highly active ^47^Sc in a nuclear reactor was described by Mausner and coworkers [8, 12]. Enriched ^47^TiO_2_ target was irradiated with high energy neutrons (E_n_ > 1 MeV) to produce ^47^Sc in the ^47^Ti(*n*,p)^47^Sc reaction. Various methods of ^47^Sc separation from metallic Ti and TiO_2_ targets, based on TBP extraction or cation and anion exchange processes, have been reported [12, 13]. Recently, to avoid the slow dissolution of the target in hot concentrated H_2_SO_4_, a new, simple and fast method based on irradiation of the Li_2_^47^TiF_6_ or ^47^TiO_2_ target, dissolution in HF solution and ion exchange isolation of ^47^Sc has been investigated [11].

As shown in Table 1, the advantage of ^47^Sc production contrary to that of ^177^Lu and ^161^Tb, is relatively easy isolation of the radionuclide from the target, but the disadvantage is smaller cross-section of the nuclear reaction.

To date, only few reports concerning labelling studies with Sc radionuclides were published [14–17] Authors of these papers concluded, that for labelling peptides, such as octreotide, the macrocyclic ligand 1,4,7,10-tetraazacyclododecane-1,4,7,10-tetraacetic acid (DOTA), is the most suitable. The stability of the DOTA complexes results from their kinetical inertness, therefore efficient labelling of DOTA-conjugates requires elevated (90 °C) temperature. Due to its rather long half-life, ^47^Sc can be also considered for preparation of alternative therapeutic agents with longer pharmacokinetics, such as ^47^Sc-labelled proteins, as e.g. monoclonal antibodies, its fragments and nanobodies. Unfortunately, because proteins denaturize at temperature above 42 °C, ligands other than DOTA, which form complexes at room temperature, are necessary for labelling such biomolecules. The acyclic chelators are not as kinetically stable as the macrocyclic chelators (DOTA, NOTA, TETA etc.), but formation of their complexes at room temperature is much faster. In this paper we report formation and stability studies on ^47^Sc complexes with various acyclic polydentate ligands, which exhibit faster than DOTA kinetics of complex formation.

## Experimental

### Materials

The analytical grade reagents were used for radiochemical investigations. Water was obtained from a Millipore water purification system. Ammonium acetate and hydrofluoric acid (40 %) were purchased from Sigma-Aldrich. All resins (Chelex^®^ 100, Dowex^®^ 1X8, Dowex^®^ 50WX4) were purchased in 100–200 mesh size from Dow Chemical.

The analytical grade TiO_2_ was obtained from Sigma-Aldrich. Enriched ^47^TiO_2_ was supplied by Isoflex, San Francisco, USA. The isotopic composition was: ^46^Ti-4.0, ^47^Ti-65.8, ^48^Ti-26.8, ^49^Ti-1.8, ^50^Ti-1.6 %.

We have chosen the following acyclic ligands for the studies: 8-dentate diethylenetriaminepentaacetic acid (DTPA), 6-dentate *N,N*-bis(2-hydroxybenzyl)ethylenediamine-*N,N*-diacetic acid (HBED), 6-dentate 1,2-bis(o-aminophenoxy)ethane-*N,N,N′,N′*-tetraacetic acid (BAPTA), 8-dentate ethylene glycol-bis(2-aminoethylether)-*N,N,N′,N′*-tetraacetic acid (EGTA), 10-dentate triethylenetetraamine-*N,N′,N′′,N′′′*-hexaacetic acid (TTHA) and deferoxamine (DFO). For comparison, cyclic 8-dentate ligand, the 1,4,7,10-tetraazacyclododecane-1,4,7,10-tetraacetic acid (DOTA) has been chosen.

### Radionuclides

For reasons of availability we used in preliminary experiments instead of ^47^Sc, the longer lived ^46^Sc radionuclide. The former is produced by fast neutron irradiation of the ^47^Ti target, while ^46^Sc can be produced in a simple way by direct thermal neutron irradiation of natural scandium. The ^46^Sc radionuclide was obtained by neutron irradiation of the Sc_2_O_3_ target at a neutron flux of 1.5 × 10^14^ n cm^−2^ s^−1^ for 200 h in the Maria research reactor in Świerk (Poland). The specific activity of the radionuclide was 3.8 GBq/mg. The target was then dissolved in 0.1 M HCl.

^47^Sc was produced by fast neutrons in the ^47^Ti(*n*,p)^47^Sc reaction on enriched (66 %) ^47^Ti target. The 2 mg samples of ^47^TiO_2_ target were irradiated in the fast neutron channel of nuclear reactor in Świerk (Poland) for 143 h at fast neutron flux (>1 MeV) of 3 × 10^13 ^n cm^−2 ^s^−1^, and thermal neutron flux of 2.5 10^14 ^n cm^−2 ^s^−1^. The irradiated ^47^TiO_2_ was then dissolved in 1 ml of concentrated hydrofluoric acid (2–4 h with gentle heating at 80 °C) and the solution was diluted to 20 ml with 1 M HF. The solution was next passed through anion exchange bed (Dowex^®^ 1X8). ^47^Sc was quantitatively eluted using the 0.04 M HNO_3_ + 0.06 M HF solution. For additional purification the eluent was evaporated to dryness and the residue was dissolved in 0.1 M HNO_3_. Afterwards ^47^Sc was adsorbed on cation exchange column Dowex^®^ 50WX4 and finally eluted in two 1 ml fractions using 0.25 M ammonium acetate.

### Syntheses of radiolabelled complexes

The experimental conditions for labelling, such as metal-to-ligand molar ratio and time of reaction were optimized to achieve high complexation yield. The ^46^Sc complexes were synthesized by mixing 164 nmol of carrier added ^46^Sc in chloride form with aqueous solutions of chelators in various molar ligand-to-metal ratios (1/1, 2/1, 5/1, 10/1). In the case of n.c.a. ^47^Sc 1 MBq was used and the ligand amount varied from 2 to 15 nmol. The complexes were prepared at room temperature at pH = 6.0 (20 mM acetate buffer). The radiolabelling yield was determined by thin layer chromatography (TLC) using silica gel plates (Polygram, Macherey–Nagel). The NH_3_/H_2_O (1/25) mixture was used as the mobile phase. The distribution of activity on paper strips was measured by cutting the paper into 1-cm pieces and counting in the NaI (Tl) well counter. The ^46^Sc-complexes moved with the solvent front (*R*_f_ = 1), while free ^46^Sc remained at the starting point.

### Stability of complexes

Stability of the ^46,47^Sc acyclic complexes in isotonic NaCl solution and PBS buffer was assessed by adding 20 μl of radiocomplex solution to 500 μl of 0.9 % NaCl and 0.01 M PBS buffer. The solutions were incubated at 37 °C and kinetics of the Sc complex dissociation was measured by taking aliquots of the NaCl and PBS solution at different time points and measuring the liberated ^46^Sc by ITLC analysis.

## Results and discussion

### Radiolabelling and kinetics

**Fig. 1:**
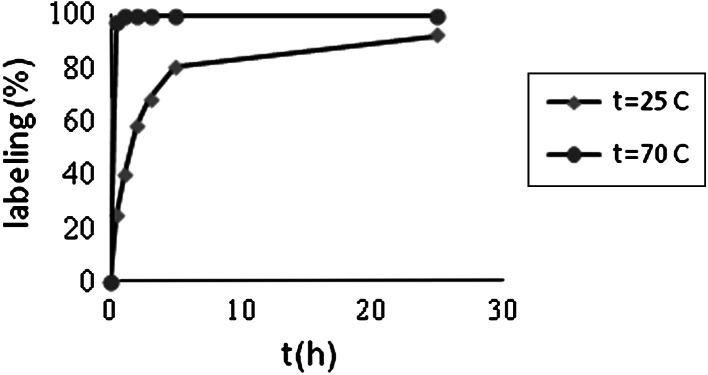
Labelling yield of the DOTA ligand with ^46^Sc at room and elevated temperature

As already mentioned, the cyclic polyamino-polycarboxylate ligands like DOTA, due to formation of kinetically inert complexes, are excellent ligands for binding M^3+^ to biomolecules. DOTA labelled with ^90^Y, ^177^Lu, ^213^Bi and ^68^Ga is widely used in peptide radiopharmaceuticals such as octreotide, bombesin, substance P etc. However, complex formation with macrocyclic DOTA derivatives generally requires, in contrast to open-chain analogues, heating at elevated temperatures (>90 °C). Because the aim of our studies was elaboration of a method suitable for labelling proteins with ^47^Sc, we studied at first the kinetics of formation of Sc-DOTA complexes at room temperature. Figure 1 presents kinetics of scandium complexation by DOTA at 25 and 70 °C.

**Fig. 2:**
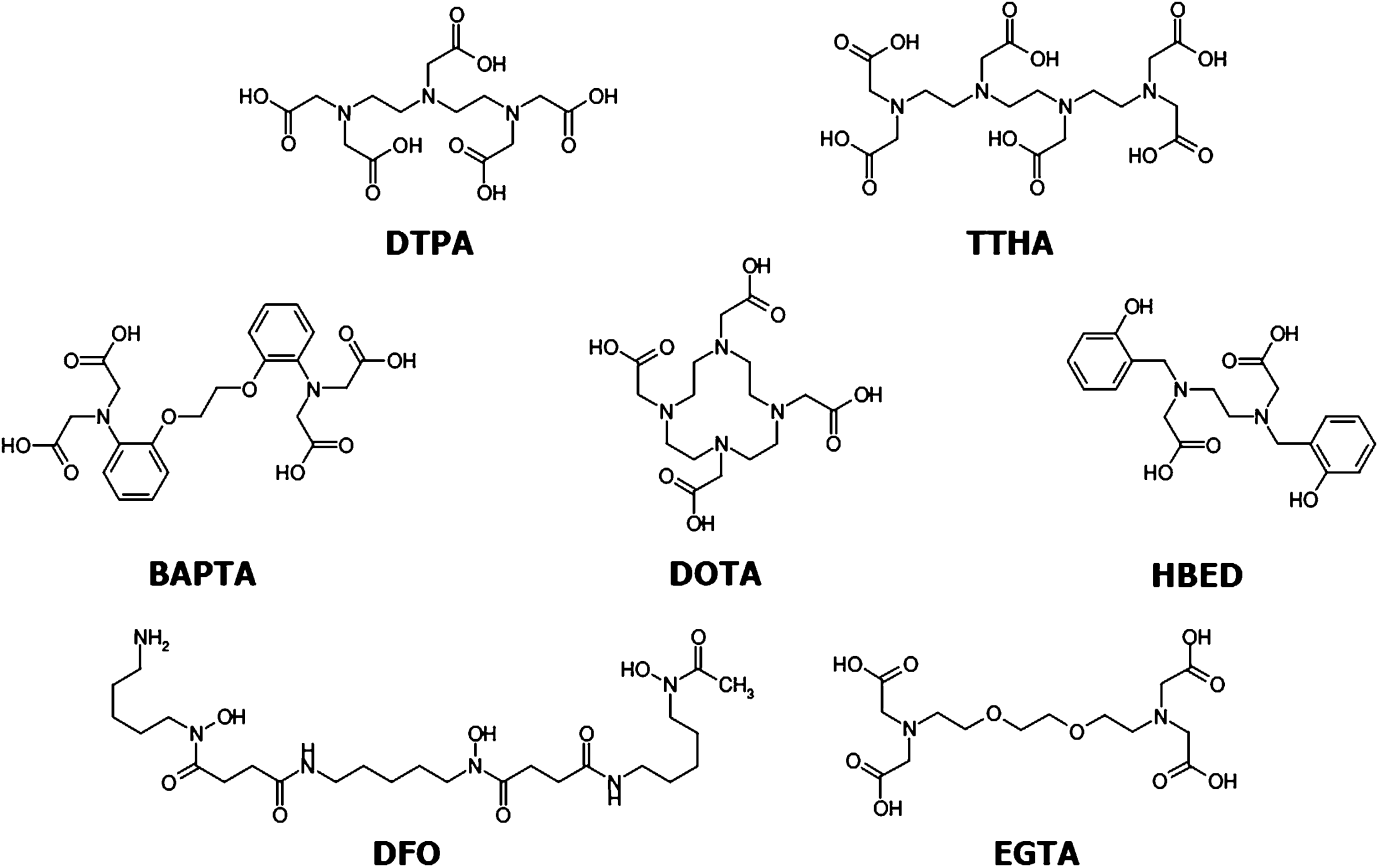
Structures of the ligands

As shown in Fig. 1 the kinetics of complexation at room temperature is slow, while increase of temperature to 70 °C drastically increases kinetics of labelling. Therefore, macrocyclic ligands, like DOTA, are not suitable for labelling of thermal non-resistant molecules, like proteins. In the present work, we examined selected acyclic polyamino-polycarboxylate ligands, which form complexes with scandium cations more rapidly than does DOTA. The ligands demonstrating high affinity for 3+ metal cations such as Fe^3+^, Ga^3+^ and lanthanides were selected for our studies. Structures of the ligands are presented in Fig. 2.

**Fig. 3:**
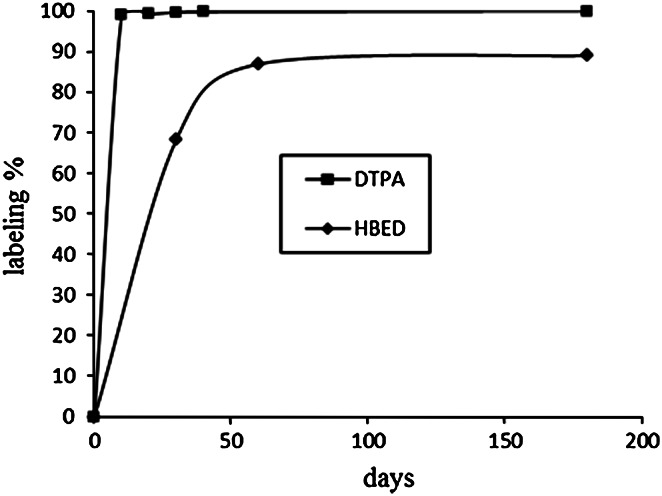
Labelling yield of DTPA and HBED ligands with ^46^Sc at room temperature

Kinetics of ^46^Sc complexation by open chain ligands was studied, in contrary to the DOTA, only at room temperature (Fig. 3). Comparison of Fig. 3. with the Fig. 1 shows much faster labelling at room temperature, of the open chain ligands than of the cyclic DOTA. In the case of DTPA the labelling yield after 10 min reached more than 99 % of the equilibrium value, while in the case of DOTA achievements of about 90 % value required more than 20 h. However, as shows in Fig. 3, the open chain ligand HBED seems, not to attain within 30 h equilibrium value.

The labelling yield of acyclic ligands with ^46^Sc was studied by us at different metal-to-ligand molar ratios, see Table 2. Our studies showed that at pH = 6.0 more than 96 % of ^46^Sc was complexed with just 1:1 ligand-to-metal molar ratio.

**Table 2:** Labelling of the ligands with carrier added ^46^Sc

Sc:ligand molar ratio (Sc = 1.6×10^−7 ^M)	Labelling yield (%)				
	EGTA	TTHA	DTPA	DFO	BAPTA
1:1	99.3	98.0	99.1	96.2	98.2
1:2	100	100	100	100	99.0
1:10	100	100	100	100	100

### In vitro stability studies

**Fig. 4:**
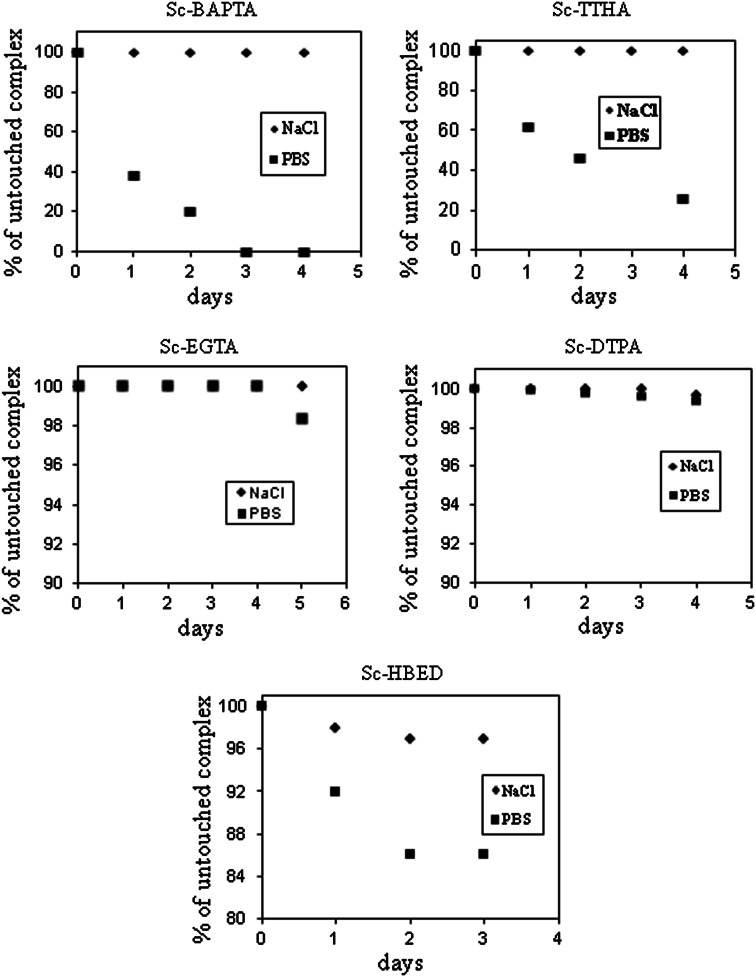
Stability of ^46^Sc-BABTA, ^46^Sc-TTHA, ^46^Sc-EGTA, ^46^Sc-DTPA and ^46^Sc-HBED complexes in 0.9 % NaCl and 0.1 M PBS buffer

For radionuclide therapy applications, radionuclides must remain associated with the targeting chelate-protein to avoid the toxicity released in their dissociation. As shown in Fig. 4, all synthesized ^46^Sc-complexes exhibited high stability in isotonic NaCl solution. After 120 h incubation in the NaCl solution more than 96 % of ^46^Sc remained in complexed form. In 0.1 M PBS buffer ^46^Sc-DTPA and ^46^Sc-EGTA complexes were stable over the whole course of the experiment. Stability of ^46^Sc-TTHA and ^46^Sc-BABTA in PBS solution was very low due to replacement of ligands by phosphates in the first coordination sphere. In the case of ^46^Sc-HBED slow decomposition of the complex was observed.

### Labelling of the ligands with n.c.a. ^47^Sc

The obtained results on complex formation and stability of ^46^Sc complexes in PBS buffer shows that only Sc-DTPA and Sc-EGTA can be used as precursors for scandium radiopharmaceuticals. Therefore, we have chosen the two ligands for further studies with n.c.a. ^47^Sc. Various concentrations of the ligands were investigated in order to evaluate their usability for binding ^47^Sc to biomolecules. Radiolabelling was performed with quantities of the ligands from 2 to 15 nmol (Table 3). For comparison, results of labelling the commonly used macrocyclic ligand DOTA [8] is presented in Table 4.

**Table 3:** Labelling of the open chain ligands with n.c.a. ^47^Sc

Amount of ligand (nmol)	Labelling yield (%)	
	DTPA	EGTA
2	38.81	97.20
5	94.89	98.80
10	95.40	99.40
15	100.0	100.0

**Table 4:** Labelling of the macrocyclic ligand with n.c.a. ^47^Sc

Amount of DOTA (nmol)	Labelling efficiency (%)
22.5	79.4
36.0	96.2
58.5	97.3

From comparison of the Table 3 with the Table 4 one can conclude that both ligands, DTPA and EGTA, form complexes with n.c.a. ^47^Sc, in lower amount of ligand than those formed by DOTA. Particularly in the case of EGTA, only 2 nmol of the ligand is sufficient to achieve labelling higher than 97 %. The reason is that EGTA and DTPA as a 8-dentate ligand, forms strong complexes by binding via four carboxylic oxygen atoms, two ether oxygen atoms and two nitrogen atoms (EGTA) [18] or five carboxylic and tree nitrogen (DTPA). As was reported by Thakur et al. [19], contrary to our results Am^3+^, Cm^3+^ and Eu^3+^ form stronger complexes with DTPA than EGTA. Formation of stronger complexes by Sc^3+^ with EGTA than DTPA is probably related with stronger interaction of smaller and harder Sc^3+^ with ether than with carboxylic oxygen atoms.

## Conclusion

In this paper formation and in vitro stability of a series of polyamino-polycarboxylate ligands labelled with ^46^Sc and ^47^Sc has been studied. We found that acyclic ligands (except HBED) form complexes much faster than the cyclic DOTA. The radiolabelling yield of acyclic ligands was very high and for the Sc:L molar ratio of 1:1 varied after 10 min from 96 to 99 %. The obtained complexes were stable in isotonic NaCl solution. However, stability of ^46^Sc-TTHA, ^46^Sc-BABTA and ^46^Sc-HBED in PBS buffer was low, due to formation by Sc^3+^stronger complexes with phosphates than with TTHA, BABTA and HBED. We have shown that when using n.c.a. ^47^Sc only 2 nmol of the EGTA is sufficient to obtain labelling yield greater than 97 %. Therefore, Sc-EGTA is a promising moiety for coupling ^47^Sc to proteins. However, biological studies on animal models are needed for evaluation the in vivo stability of radiometal-labelled chelates.

## References

[1] Firestone RB, Shirley VS, Baglin CM, Chu SYF, Zipkin J (1996). Table of isotopes.

[2] Lebedev NA, Novgorodov AF, Misiak R, Brockmann J, Rösch F (2000). Radiochemical separation of no-carrier-added ^177^Lu as produced *via* the ^176^Yb(*n,γ*)^177^Yb → ^177^Lu process. Appl Radiat Isot.

[3] Hashimoto K, Matsuoka H, Uchida S (2003). Production of no-carrier added ^177^Lu *via* the ^176^Yb(*n,γ*)^177^Yb → ^177^Lu process. J Radioanal Nucl Chem.

[4] Balasubramanian PS (1994). Separation of carrier-free lutetium-177 from neutron irradiated natural ytterbium target. J Radioanal Nucl Chem.

[5] Horwitz EP, McAlister DR, Bond AH, Barrans RE, Williamson JM (2005). A process for the separation of ^177^Lu from neutron irradiated ^176^Yb targets. Appl Radiat Isot.

[6] Bilewicz A, Żuchowska K, Bartoś B (2009). Separation of Yb as YbSO_4_ from the ^176^Yb target for production of ^177^Lu via the ^176^Yb(*n,*γ)^177^Yb → ^177^Lu process. J Radioanal Nucl Chem.

[7] Lehenberger S, Barkhausen Ch, Cohrs S, Fischer E, Grünberg J, Hohn A, Köster U, Schibli R, Türler A, Zhernosekov K (2011). The low-energy β− and electron emitter ^161^Tb as an alternative to ^177^Lu for targeted radionuclide therapy. Nucl Med Biol.

[8] Mausner LF, Kolsky KL, Joshi V, Srivastava SC (1998). Radionuclide development at BNL for nuclear medicine therapy. Appl Radiat Isot.

[9] Kopecky P, Szelecsenyi F, Molnàir T, Mikecz P, Tfirkànyi F (1993). Excitation functions of (*p,xn*) reactions on ^nat^Ti: monitoring of bombarding proton beams. Appl Radiat Isot.

[10] Bokhari TH, Mushtaq A, Khan IU (2009). Separation of no-carrier-added radioactive scandium from neutron irradiated titanium. J Radioanal Nucl Chem.

[11] Bartoś B, Majkowska A, Krajewski S, Bilewicz A (2012). New separation method of no-carrier-added ^47^Sc from titanium targets. Radiochim Acta.

[12] Pietrelli L, Mausner LF, Kolsky KL (1992). Separation of carrier free ^47^Sc from titanium targets. J Radioanal Nucl Chem.

[13] Kolsky KL, Joshi V, Mausner LF, Srivastava SC (1998). Radiochemical purification of no-carrier-added scandium-47 for radioimmunotherapy. Appl Radiat Isot.

[14] Mausner LF, Joshi V, Kolsky KL, Meinken GE, Mease RC, Sweet MP, Srivastava SC (1995). Evaluation of chelating agents for radioimmunotherapy with scandium-47. J Nucl Med.

[15] Majkowska-Pilip A, Bilewicz A (2011). Macrocyclic complexes of scandium radionuclides as precursors for diagnostic and therapeutic radiopharmaceuticals. J Inorg Biochem.

[16] Pruszynski M, Majkowska A, Loktionova NS, Roesch F (2012). Radiolabeling of DOTATOC with the longer-lived, generator derived positron emitter ^44^Sc. Appl Radiat Isot.

[17] Huclier-Markai S, Sabatie A, Ribet S, Kubıcek V, Paris M, Vidaud C, Hermann P, Cutler CS (2011). Chemical and biological evaluation of scandium(III)–polyaminopolycarboxylate complexes as potential PET agents and radiopharmaceuticals. Radiochim Acta.

[18] Inomata Y, Okamura D, Morita K, Yukawa Y, Howell FS (2003). The comparison of crystal structures of lanthanide complexes with 3,12-bis(carboxymethyl)-6,9-dioxa-3,12-diazatetradecanedioic acid (H_4_egta). J Mol Struct.

[19] Thakur P, Conca JL, Choppin GR (2011). Complexation studies of Cm(III), Am(III), and Eu(III) with linear and cyclic carboxylates and polyaminocarboxylates. J Coord Chem.

